# Mechanism-of-Action Classification of Antibiotics by Global Transcriptome Profiling

**DOI:** 10.1128/AAC.01207-19

**Published:** 2020-02-21

**Authors:** Aubrie O’Rourke, Sinem Beyhan, Yongwook Choi, Pavel Morales, Agnes P. Chan, Josh L. Espinoza, Chris L. Dupont, Kirsten J. Meyer, Amy Spoering, Kim Lewis, William C. Nierman, Karen E. Nelson

**Affiliations:** a Department of Human Biology, J. Craig Venter Institute, La Jolla, California, USA; b Department of Genomic Medicine, J. Craig Venter Institute, La Jolla, California, USA; c Department of Human Biology, J. Craig Venter Institute, Rockville, Maryland, USA; d Department of Genomic Medicine, J. Craig Venter Institute, Rockville, Maryland, USA; e Department of Biology, Antimicrobial Discovery Center, Northeastern University, Boston, Massachusetts, USA; f NovoBiotic Pharmaceuticals, Cambridge, Massachusetts, USA

**Keywords:** antimicrobials, antibiotics, drug discovery, *E. coli*, transcriptomics, mechanism of action, dereplication

## Abstract

Antimicrobial resistance (AMR) is an ever-growing public health problem worldwide. The low rate of antibiotic discovery coupled with the rapid spread of drug-resistant bacterial pathogens is causing a global health crisis. To facilitate the drug discovery processes, we present a large-scale study of reference antibiotic challenge bacterial transcriptome profiles, which included 37 antibiotics across 6 mechanisms of actions (MOAs) and provide an economical approach to aid in antimicrobial dereplication in the discovery process. We demonstrate that classical MOAs can be sorted based upon the magnitude of gene expression profiles despite some overlap in the secondary effects of antibiotic exposures across MOAs. Additionally, using gene subsets, we were able to subdivide broad MOA classes into subMOAs. Furthermore, we provide a biomarker gene set that can be used to classify most antimicrobial challenges according to their canonical MOA. We also demonstrate the ability of this rapid MOA diagnostic tool to predict and classify the expression profiles of pure compounds and crude extracts to their expression profile-associated MOA class.

## Abstract (precis)

Antimicrobial resistance (AMR) is an ever-growing public health problem worldwide. The low rate of antibiotic discovery coupled with the rapid spread of drug-resistant bacterial pathogens is causing a global health crisis. To facilitate the drug discovery processes, we present a large-scale study of reference antibiotic challenge bacterial transcriptome profiles, which included 37 antibiotics across 6 mechanisms of actions (MOAs) and provide an economical approach to aid in antimicrobial dereplication in the discovery process.

## INTRODUCTION

The Centers for Disease Control and Prevention estimates that 2 million individuals in the United States will acquire an antimicrobial resistance (AMR) infection annually, leading to 23,000 expected mortalities (1). Globally, it is predicted that AMR infections will lead to the deaths of 10 million people/year by 2050 (2). At present, treatment options are limited and overused; thus, the search for new antibiotics with novel chemistry using methods less costly than traditional analytical chemistry techniques alone is critical. Transcriptomics can meet this need through the rapid binning of global gene expression patterns into the following six classical mechanism of action (MOA) categories: inhibitors of DNA replication (DNA synthesis [DS] and DNA gyrase [DG]), RNA synthesis [RS], protein synthesis (50S [PS50] or 30S [PS30] subunit inhibitors), cell wall biosynthesis (CW), cell membrane biosynthesis (CM), and fatty acid synthesis (FAS). Using transcriptome profiling, novel compounds or extracts, which sort to the desired target MOA, can be further investigated for novel chemistry using more costly analytical chemistry techniques.

The current understanding of the biological activity of antibiotics is largely centered on their primary cellular targets. However, when viewing an antibiotic-treated transcriptome, the signature obtained is less that of genes associated with the direct effects of inhibiting the primary protein target and more representative of the downstream secondary effects of antibiotic treatment (3). These secondary effects are often convergent and are involved in a response to stress and the regulation of biochemical pathways (4). As a result, antimicrobials with different MOAs have overlapping differentially expressed genes (DEGs). Until now, there have been two large-scale transcriptomic investigations that have provided a starting point for deconvoluting antibiotic-treatment transcriptomes and predicting MOAs based upon the differential gene expression profiles of Bacillus subtilis (5) and Mycobacterium tuberculosis (6). Both studies used microarray technology to provide a high-throughput pipeline alternative to analytical chemistry-based dereplication techniques. In this study, we use next-generation sequencing technology to determine the transcriptomic signatures of E. coli in response to 37 antibiotics with known mechanisms and predict the MOA of novel antimicrobials based on expression profiling. This standardized approach can be implemented in any laboratory setting at a low cost to provide useful MOA data, preceding costlier chemical analysis.

## RESULTS

**FIG 1:**
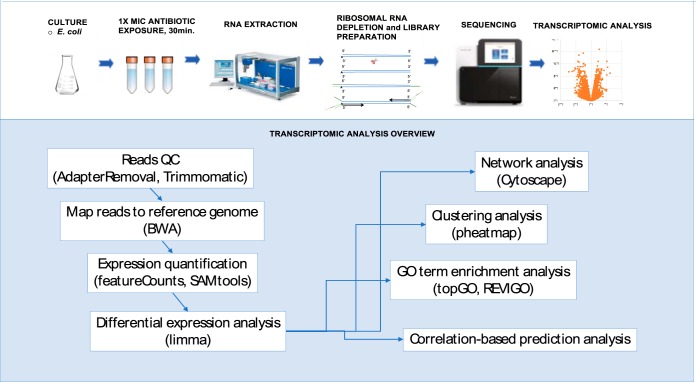
Sample processing overview.

Historically, Gram-positive bacteria were used in high-throughput antimicrobial screens because they do not have the hard-to-penetrate outer cell membrane that is characteristic of Gram-negative species (7). To overcome this limitation in a screening approach with a Gram-negative bacterium, we used the efflux-deficient Escherichia coli WO153 (parent strain AB1157; with additional mutations *asmB1* Δ*tolC*::*kan*) (https://cgsc2.biology.yale.edu/Strain.php?ID=4509) which is a strain with a compromised outer membrane (8). This allowed us to observe an effect on the normally impervious Gram-negative cells. The E. coli WO153 strain was treated with each of the 37 compounds at a 1× MIC (Table 1) for 30 minutes, a time at which >60% of the population remains viable. We used optical density at 600 nm (OD_600_) values of the treatment compared to a solvent control as a rapid means to assess relative survivorship and a proxy for viability (see Fig. S1 in the supplemental material). Subsequently, transcriptome sequencing (RNA-seq) library preparation, sequencing, and analysis were performed on 219 samples (Fig. 1; see Data Set S1 in the supplemental material).

**TABLE 1:** List of antibiotics^*a*^ used in this study

Inhibitor by type	MIC (μg/ml) in MHBII^*b*^	Antibiotic class(es)	Total no. of DEGs	No. of upregulated DEGs	No. of downregulated DEGs
Cell membrane					
Valinomycin	400	Depsipeptides	650	446	204
Polymyxin	0.0625	Lipopeptides	48	45	3
Monactin	4	Macrotetrolide	1,500	751	749
Colistin	0.125	Lipopeptides	0	0	0
Cell wall synthesis					
Vancomycin	64	Glycopeptides	309	233	76
Teixobactin	2.5	Depsipeptide	173	63	110
Ramoplanin	8	Glycolipodepsipeptide	347	218	129
Phosphomycin	16	Organic phosphonic acid	113	60	53
penicillin G	16	Beta-lactams (penicillins)	104	81	23
Meropenem	0.25	Beta-lactams (carbapenem)	112	57	55
Flavomycin	2	Phosphoglycolipids	172	96	76
D-cycloserine	64	Analog of d-alanine	0	0	0
Ceftriaxone	0.015625	Beta-lactams (cephalosporins), 3rd generation	13	11	2
Cefotaxime	0.03125	Beta-lactams (cephalosporins), 3rd generation	9	6	3
Bacitracin	125	Peptides	828	393	435
DNA gyrase					
Novobiocin	0.5	Coumarin-glycosides	1,899	892	1,007
Norfloxacin	0.015626	Fluoroquinolone	17	17	0
Naldixic acid	1	Quinolone	12	10	2
Ciprofloxacin	0.003906	Fluoroquinolone	25	21	4
DNA synthesis					
Trimethoprim	0.25	Pyridine/pyrimidine	396	143	253
Sulfamethoxazole	400	Sulfonamides	74	25	49
Metronidazole	800	Nitroimidazole	398	109	289
Doxorubicin	0.5	Anthracycline	691	373	318
Fatty acid synthesis					
Triclosan	0.007813	Chlorophenol	929	463	466
Isoniazid	900	Pyridinecarboxylic acids	294	143	151
Cerulenin	16	Oxirane carboxylic acids	627	238	389
Protein synthesis (30S ribosomal subunit)					
Tetracycline	0.5	Tetracyclines	1,050	544	506
Doxycycline	0.125	Tetracyclines	404	109	295
Protein synthesis (50S ribosomal subunit)					
Thiostrepton	8	Thiopeptides	1,354	565	789
Retapamulin	0.015625	Pleuromutilin	1,718	796	922
Linezolid	4	Oxazolidinones	1,072	420	652
Erythromycin	0.25	Macrolides	1,497	663	834
Chloramphenicol	1	Nitrobenzenes, amphenicols	1,755	858	897
RNA synthesis					
Rifapentine	1	Rifamycins	2,106	1,004	1,102
Rifampin	0.0625	Rifamycins	836	585	251
Fidaxomicin	0.125	Macrocyclic	6	6	0
Actinomycin D	32	Cyclic depsipeptides	1,850	851	999

a Presented with MIC values and number of DEGs with |log2(FC)| of >1 and FDR of <0.01 as the cutoffs.

b MHBII, cation-adjusted Mueller-Hinton II broth.

Upon filtering with a >2-fold change in transcript level and <1% false discovery rate (FDR), a method for identifying the rate of type I errors in null hypothesis testing when conducting multiple comparisons, we observed a large range (average of 668, standard deviation [stdev.] of ±661) in the number of genes differentially expressed in response to antibiotic treatment irrespective of MOA designation. Colistin and d-cycloserine did not yield a significant number of DEGs due to the high FDR values (Table 1; see Fig. S2 in the supplemental material; see Data Set S2 in the supplemental material) and, thus, were not included in portions of the analyses that require fold change cutoffs backed by a defined FDR, but they were included in further predictive modeling.

**FIG 2:**
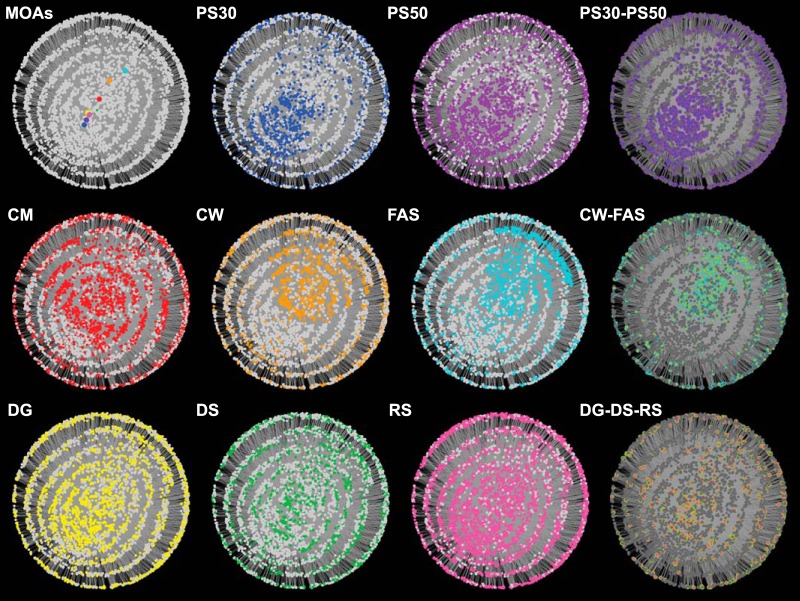
Network view of the 3,852 DEGs generated using the edge-weighted spring-embedded network in Cytoscape v3.7. Nodes representing DEGs and MOAs were colored according to the MOA, as follows: CM, red; CW, orange; DG, yellow; DS, green; FAS, cyan; PS30, blue; PS50, purple; and RS, pink.

To illustrate the underlying MOA-specific expression profiles amid transcriptomic convergence, a network visualization of DEGs of compounds within each MOA was generated using the Cytoscape program (9) (Fig. 2). The edge-weighted spring-embedded layout in the Cytoscape platform was chosen to create the self-organized network. This algorithm was initially generated by Kamada and Kawai to organize undirected network structures (10). Based on this algorithm, each node is subject to forces that are applied by the edges that act similar to metal springs. Edges were defined as their presence or absence in each MOA. We additionally used edge weights which were calculated as a ratio of the number of compounds that each DEG was detected over the number of total of compounds that were included in a given MOA (see Data Set S3 in the supplemental material). These edge weights determined the magnitude of forces applied on each node in the network.

Out of 4,140 protein-coding genes, there were 3,852 DEGs with a >2-fold change in transcript level and <1% FDR represented in at least one treatment (see Fig. S3 in the supplemental material; Data Set S3). These cutoffs were selected to be inclusive to capture the differing levels of DEGs across antibiotic treatments. In total, the network was composed of 3,860 nodes (3,852 gene nodes and 8 MOA nodes due to the separation of the DS/DG and PS30/PS50 subMOA group) and 14,205 edges. The placement of 8 MOA nodes within the network illustrates that the global transcriptomic responses of the CW-FAS, PS30-PS50, and DG-DS-RS split according to similarity (Fig. 2). An overlay of MOA-specific coloring to the network reveals the striking similarity between specific groups, where 31.1% (601) of the DEGs were shared between the FAS and CW inhibitor groups and 44.5% (1,137) of the nodes were shared between the PS30 and PS50 inhibitor groups (Fig. 2). DS, DG, and RS inhibitor groups displayed a dispersed pattern, with 534 DEGs shared among the three MOAs. In addition, there were 445 DEGs that were significant in only 1 MOA classification (Data Set S3). Overall, our results suggest that while there are shared expression patterns across certain MOA classifications, MOA-specific patterns do exist.

**FIG 3:**
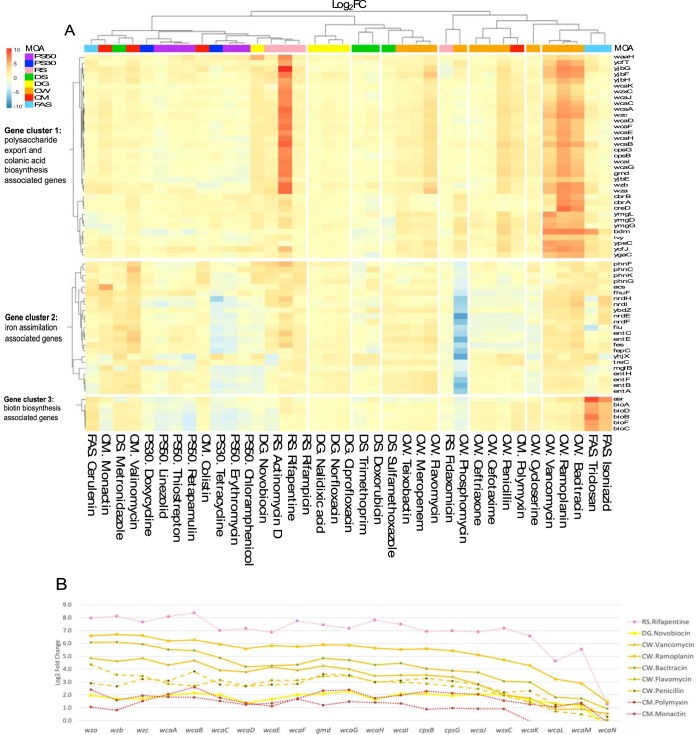
Unsupervised clustering. (A) Hierarchical clustering analysis of 37 antimicrobial-induced transcriptomes identified three distinct gene signatures. Gene expression fold change values (log_2_) are shown with genes as rows and antimicrobial compounds as columns. The three distinct signatures are colanic acid gene cluster (top), iron-assimilation genes (middle), and biotin synthesis gene cluster (bottom). (B) Colanic acid-associated gene expression is shown in log_2_FC values across the *wca* gene cluster for antimicrobial compounds ramoplanin (CW), vancomycin (CW), bacitracin (CW), flavomycin (CW), polymyxin (CM), monactin (CM), rifapentine (RS), and novobiocin (DG).

To further evaluate the MOA-specific and convergent trends in the data set, an unsupervised clustering of the 37 antibiotic profiles (including colistin and cycloserine) was performed. This approach again demonstrates that antibiotic treatments do not cluster exclusively according to their MOA designation (Fig. 3A; see Fig. S4 in the supplemental material). However, three expression signatures were identified, namely, two for cell wall inhibitor compounds and one for FAS inhibitors. The magnitude of expression for the transcripts of one such cluster was able to discriminate between MOA classes (Fig. 3A). This pronounced expression signature had an over 30-fold upregulation across a group of cell wall inhibitor compounds, namely, ramoplanin, bacitracin, and vancomycin (Fig. 3A, gene cluster 1; see Data Set S4 in the supplemental material). These compounds belong to the Lipid II cycle inhibitor subgroup, which block peptidoglycan synthesis through substrate sequestration and tight binding to Lipid II at the extracellular surface of the cytoplasmic membrane (11). As many as 30% of the upregulated genes are members of the *wca* gene cluster (locus ID: b2042-b2062) with a role in polysaccharide export and colanic acid biosynthesis (12). Additional members of this first gene expression signature included *yjbFG* (b4027 and b4028), *ymgDG* (b1171 and b1172), and *cbrABC* (b3690, b3716, and b3717).

To assess the potential use of the *wca* genes as biomarkers in high-throughput screening for cell wall inhibitor activities, a comparison of the capsule synthesis *wca* gene expression per antimicrobial treatment was made (Fig. 3B; see Fig. S5 in the supplemental material; Data Set S4). In addition to the strong upregulation found in ramoplanin, bacitracin, and vancomycin, moderate upregulation was detected in the following three additional cell wall inhibitors: flavomycin (Lipid II transglycosylation), penicillin (transpeptidation), and meropenem (transpeptidation). The *wca* loci, however, is not diagnostic of all CW compounds; the CW compounds ceftriaxone and cefotaxime did not induce upregulation. Additionally, outside the cell wall group, upregulation of the *wca* operon was observed in polymyxin (CM), monactin (CM), novobiocin (DG), actinomycin D (RS), and rifapentine (RS).

The second profile observed was a unique cell wall gene signature in phosphomycin (Fig. 3A, gene cluster 2; Data Set S4). Phosphomycin blocks cell wall synthesis through inhibition of the enzymatic conversion of the peptidoglycan precursor UDP-*N*-acetylglucosamine to UDP-*N*-acetyl-muramic acid (13). A massive downregulation (90-fold) of iron assimilation genes, including enterobactin biosynthesis (*ent*) and transport/export (*fep*), was detected. Genes involved in nucleotide metabolism (*nrdEFH*) and a membrane protein (*yhjX*) were also strongly downregulated. The phosphomycin gene signature is highly specific to phosphomycin, although a modest level of downregulation for this gene set was observed with erythromycin (PS50; 5-fold) and tetracycline (PS30; 8-fold).

The third observed signature was shared among the fatty acid synthesis inhibitors. Triclosan and isoniazid both block the reduction step of the fatty acid synthesis pathway by inhibiting an enoyl-ACP reductase (*fabI*), whereas cerulenin inhibits a β-ketoacyl-ACP synthase (*fabB*) (14). The *bioABFCD* genes involved in biotin biosynthesis were upregulated in triclosan (83-fold) and isoniazid (21-fold) and to a lesser degree in cerulenin (6-fold) treatments (Fig. 3A, gene cluster 3; Data Set S2). In E. coli, fatty acid synthesis is carried out to assemble the pimelate precursor for entering the biotin synthetic pathway (15), where biotin is a cofactor crucial for key enzymes in metabolic and cellular processes (16). Here, the biotin gene cluster was upregulated by 3-fold in metronidazole (DS), bacitracin (CW), valinomycin (CM), and polymyxin (CM) and was not detected in the remaining antimicrobial compounds.

**FIG 4:**
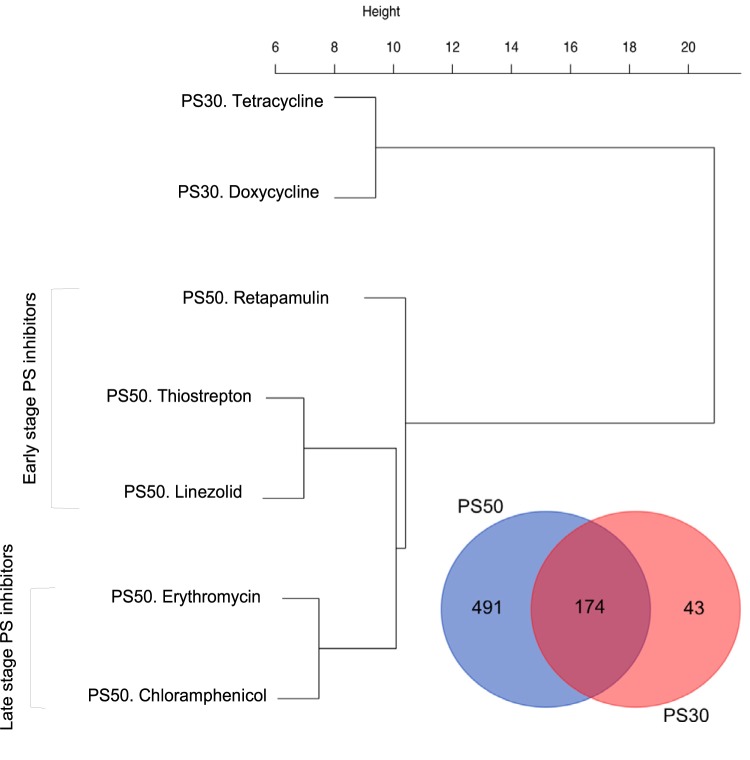
MOA-specific patterns. Venn diagram of the overlapping DEG for the PS50 and PS30 overlap, and hierarchical clustering of 174 overlapping DEG genes for the PS50 and PS30 overlap.

Next, we performed the first of the two supervised approaches to assess whether antibiotics within a given MOA can be further classified into subMOAs based upon DEG patterns. We present two examples here and provide the overlap and clustering results for the DEGs of all MOA subgroups in Fig. S6 and S7 and Data Set S5 in the supplemental material. The five PS50 antibiotics (thiostrepton, retapamulin, linezolid, erythromycin, and chloramphenicol) and two PS30 antibiotics (tetracycline and doxycycline) could be stratified by the magnitude of their transcriptomic response for a subset of 174 DEGs common to the subMOAs (Fig. 4). Furthermore, the 50S inhibitors clustered according to their role as early- or late-stage protein synthesis inhibitors (Fig. 4). In this 174-gene list, a majority of the upregulated transcripts were associated with either the 50S or the 30S ribosomal subunit (Data Set S5).

**FIG 5:**
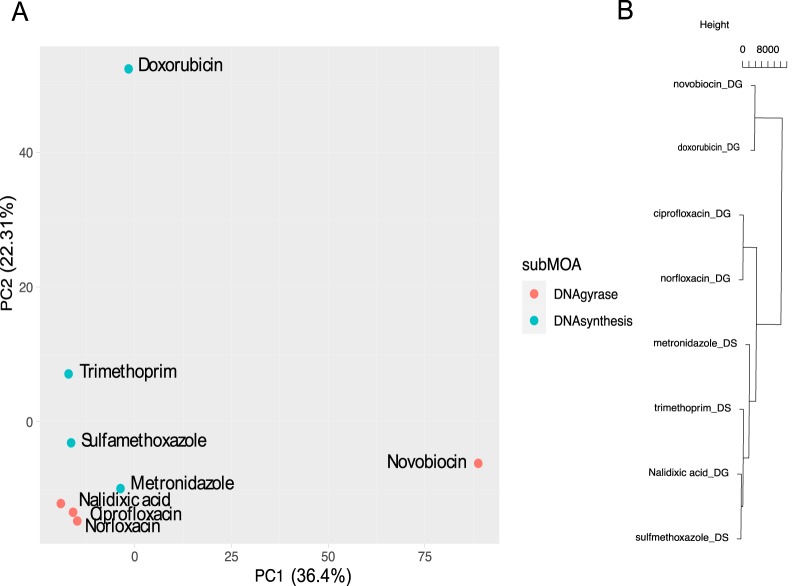
MOA-specific patterns. (A) PCA of all DEGs for DNA synthesis/DNA gyrase and (B) hierarchical clustering of the molecular descriptors for DS/DG inhibitors (below).

Unlike the PS data set, no DEGs were shared across the eight DS-inhibiting antibiotics. However, principal-component analysis (PCA) of the union set of genes (Data Set S5) shows that the transcriptomic responses will split according to whether the antibiotic is a DNA gyrase/topoisomerase IV inhibitor or acts indirectly to inhibit DNA synthesis (Fig. 5A). Novobiocin and doxorubicin appear as transcriptomic outliers within their respective subMOA. Novobiocin is the only DG inhibitor in our data set which targets the DNA gyrase subunit B rather than subunit A, whereas doxorubicin is a very potent DNA intercalator, unlike the other DNA-binding or nucleotide synthesis inhibitors in our data set. It is also a potent reduction/oxidation (redox) agent that forms reactive oxygen species. These two antibiotics also have the highest number of DEGs, with 1,899 and 691 DEGs for the MOA (Table 1). The observed transcriptomic response could also be explained by the chemistries of these compounds (Fig. 5B; see Data Set S6 in the supplemental material).

Lastly, to evaluate the use of transcriptomic signatures to predict the MOA of an unknown compound, we evaluated a simple prediction model using correlation. In this model, a Pearson correlation (r) value is computed between the unknown compound and each of the reference compounds based on gene expression values of a selected set of diagnostic genes. The MOA of the unknown compound is predicted as the MOA of the reference compound with the highest correlation value. We explored three different approaches for selecting diagnostic genes, which are regulated differently either at the compound level (approaches 1 and 2) or the MOA level (approach 3). The three approaches were as follows: (1) select genes that have the highest variance across 37 compounds, (2) select genes that are the most significantly up/downregulated in each of 37 compounds, and (3) select genes whose fold change values are the most different between MOAs based on ANOVA analysis, which was used in a previous study (17) (details in Materials and Methods section). Based on the overall prediction accuracy, approach 2 showed the best performance (84% accuracy, i.e., 31 compounds with correct MOA prediction out of 37) compared with approaches 1 (70%) and 3 (81%). This could be because the set of genes collected by approach 2 better represents the unique transcriptome profile of every compound in our data set. This best-performing approach used a combined list of the top 20 most significantly differentially expressed genes collected from each compound (i.e., genes with the smallest FDR while requiring the FDR of <0.1% and not limited by an arbitrary fold change cutoff) (see Data Set S7 in the supplemental material). Hierarchical clustering of the 447-gene diagnostic set better resolved the antibiotics into their classical MOA designations (see Fig. S8 in the supplemental material). We validated the gene selection approach using a leave-one-compound-out cross-validation method (details in Materials and Methods section). The predicted MOA for the individual antimicrobial compounds and accuracy for each MOA are shown in Table 2. Here, we achieved 100% prediction accuracy for the protein synthesis, fatty acid synthesis, and cell wall inhibitors (see Fig. S9 in the supplemental material).

**TABLE 2:** MOA predictions for pure compounds and crude extracts^*a*^

Sample type	MOA or compound dereplicated	Test compound or extract	Closest compound	Pearson correlation coefficient	Predicted MOA	Same as known MOA	Reported MOA of chemically dereplicated compound
References	RNA synthesis	RS.Rifapentine	RS.Rifampin	0.71	RS	Yes	
		RS.Rifampin	RS.Rifapentine	0.72	RS	Yes	
		RS.Fidaxomicin	CW.Phosphomycin	0.18	CW	No	
		RS.ActinomycinD	RS.Rifapentine	0.67	RS	Yes	
	Protein synthesis	PS50.Thiostrepton	PS50.Retapamulin	0.95	PS	Yes	
		PS50.Retapamulin	PS50.Thiostrepton	0.95	PS	Yes	
		PS50.Linezolid	PS50.Thiostrepton	0.93	PS	Yes	
		PS50.Erythromycin	PS50.Chloramphenicol	0.92	PS	Yes	
		PS50.Chloramphenicol	PS50.Erythromycin	0.92	PS	Yes	
		PS30.Tetracycline	PS50.Erythromycin	0.88	PS	Yes	
		PS30.Doxycycline	PS50.Thiostrepton	0.84	PS	Yes	
	Fatty acid synthesis	FAS.Triclosan	FAS.Cerulenin	0.71	FAS	Yes	
		FAS.Isoniazid	FAS.Triclosan	0.56	FAS	Yes	
		FAS.Cerulenin	FAS.Triclosan	0.76	FAS	Yes	
	DNA synthesis	DS.Trimethoprim	PS30.Doxycycline	0.72	PS	No	
		DS.Sulfamethoxazole	DS.Metronidazole	0.64	DS	Yes	
		DS.Metronidazole	PS30.Doxycycline	0.68	PS	No	
		DS.Doxorubicin	DS.Trimethoprim	0.54	DS	Yes	
	DNA gyrase	DG.Novobiocin	RS.Rifapentine	0.56	RS	No	
		DG.Norfloxacin	DG.Ciprofloxacin	0.92	DG	Yes	
		DG.Nalidixic_acid	DG.Ciprofloxacin	0.84	DG	Yes	
		DG.Ciprofloxacin	DG.Norfloxacin	0.92	DG	Yes	
	Cell wall	CW.Vancomycin	CW.Ramoplanin	0.90	CW	Yes	
		CW.Teixobactin	CW.Meropenem	0.85	CW	Yes	
		CW.Ramoplanin	CW.Bacitracin	0.95	CW	Yes	
		CW.Phosphomycin	CW.Penicillin	0.30	CW	Yes	
		CW.Penicillin	CW.Vancomycin	0.76	CW	Yes	
		CW.Meropenem	CW.Flavomycin	0.88	CW	Yes	
		CW.Flavomycin	CW.Meropenem	0.91	CW	Yes	
		CW.Cycloserine	CW.Teixobactin	0.47	CW	Yes	
		CW.Ceftriaxone	CW.Cefotaxime	0.84	CW	Yes	
		CW.Cefotaxime	CW.Ceftriaxone	0.85	CW	Yes	
		CW.Bacitracin	CW.Ramoplanin	0.95	CW	Yes	
	Cell membrane	CM.Valinomycin	CM.Colistin	0.57	CM	Yes	
		CM.Polymyxin	CW.Penicillin	0.69	CW	No	
		CM.Monactin	RS.ActinomycinD	0.65	RS	No	
		CM.Colistin	CM.Valinomycin	0.59	CM	Yes	
Crude extracts from antimicrobial-producing bacterial strains	Cell membrane	CM.VALINOMYCIN.producer	CM.Monactin	0.88	CM	Yes	
	Cell wall	CW.BACITRACIN.producer	CM.Valinomycin	0.75	CM	No	
		CW.PENICILLIN.G.producer	CW.Vancomycin	0.84	CW	Yes	
		CW.TEIXOBACTIN.producer	CW.Teixobactin	0.59	CW	Yes	
		CW.VANCOMYCIN.producer	CM.Monactin	0.86	CM	No	
	DNA gyrase	DG.NOVOBIOCIN.producer	DG.Novobiocin	0.84	DG	Yes	
	Protein synthesis	PS50.CHLORAMPHENICOL.producer	PS50.Chloramphenicol	0.73	PS	Yes	
		PS.50.ERYTHROMYCIN.producer	PS50.Chloramphenicol	0.78	PS	Yes	
	RNA synthesis	RS.ACTINOMYCIN.D.producer	RS.ActinomycinD	0.79	RS	Yes	
		RS.Rifampin.producer	RS.Rifampin	0.82	RS	Yes	
Crude extracts with a prior confirmed antimicrobial activity	Desotamide	NB_E_0263	RS.ActinomycinD	0.54	RS		RS
	Streptonigrin	NB_E_0328	DG.Ciprofloxacin	0.4	DG		DS
	Nybomycin	NB_E_0196	DS.Doxorubicin	0.28	DS		DG
	LI-F05 series and Polymyxin	NB_E_0275	FAS.Cerulenin	0.49	FAS		CM
	Darobactin	ADC56	CW.Penicillin	0.66	CW		CM

a Correlation-based prediction results for antimicrobial reference compounds by leave-one-compound-out cross-validation and correlation-based prediction results for crude extracts of antimicrobial-producing microbial strains.

Our MOA predictive model based on the 447-gene diagnostic set (Data Set S7) was further tested using the transcriptome profiles of E. coli WO153 exposed to crude extracts from bacterial strains producing antimicrobials in the training set. The overall accuracy for the crude extract validation set was 80%, with an average Pearson correlation coefficient of 0.79 (Table 2). The incorrect predictions were between the highly related CW and CM MOAs. Specifically, we observed that the two cell wall inhibitors (vancomycin and bacitracin) were predicted to the cell membrane MOA.

As a final experiment, we took the transcriptomic profiles for five crude extracts and one pure compound with a prior confirmed antimicrobial activity but unknown MOA and queried them against our predictive model. Independently, chemical dereplication was used to identify the active compounds within these crude extracts, and none of these compounds were included in the training set. Generally, the model failed to make predictions with a Pearson correlation coefficient greater than 0.5 for these compounds (3 out of 5) (Table 2). In addition, the crude extract containing ADC56 was assigned to CW instead of CM, a common problem with the existing model. ADC56, also known as darobactin, has recently been described as a BamA inhibiter, where BamA is an essential outer membrane protein (18). However, the crude extract containing desotamide was accurately predicted to be an RS inhibitor, albeit with a low correlation coefficient (0.54) (Table 2).

## DISCUSSION

The ability of transcriptomic profiling to provide an antibiotic fingerprint has inspired multiple studies to use the transcriptomic profiles of bacterial strains challenged with antimicrobial compounds to avoid the rediscovery of known compounds. Prior to the current study, the largest transcriptome screening panels involved antibiotic treatments of B. subtilis and M. tuberculosis (5, 6). Both studies used the microarray platform to evaluate a transcriptomic response to antibiotic treatment. Experimentation in the Gram-positive bacterium B. subtilis demonstrated the predictive power of transcriptomic signatures. This study also used the transcriptomic profiles of 37 antibiotics within 6 MOAs to demarcate antibiotic MOA classes from one another and allowed for a blind prediction (<80% accuracy) of what class an antibiotic belonged to by transcriptomic response alone (5). Similar experiments in M. tuberculosis demonstrated that antibiotics, which fall under a particular MOA, could be identified by a subset of coordinately regulated gene clusters (6). Furthermore, they found that the expression signature of the pure compound was similar to that of the crude extract obtained from the producer organism for that compound. An RNA-seq study in Staphylococcus aureus concluded that such profiling methods could discriminate between the bacteriostatic and bactericidal compounds within the same MOA class (17). These studies suggest that it is possible to prioritize crude extracts harboring novel compounds over crude extracts harboring known compounds without the need for prior fractionation or characterization by analytical chemistry or biochemical techniques. Therefore, expression profiling is invaluable to the antibiotic discovery process as it simultaneously allows for dereplication and provides a detailed profile of the mechanism of action.

Here, we provide the largest and most comprehensive data set to use RNA sequencing technology for the purpose of characterizing the bacterial response to antibiotic treatment. We present a standardized approach which uses the efflux-deficient, outer membrane-compromised E. coli strain WO153 to build our reference data set, as it represents a composite of both the Gram-negative and Gram-positive prokaryotic cell types from the perspective of antibiotic access to the cell interior. Due to the unique background of this test strain, it is likely that some of the results, like diagnostic genes, are not transferable to experiments in different strains. In our study, a 1× MIC challenge condition was selected with a 30-minute exposure to observe the compound-specific response of each antibiotic while avoiding the indirect effects of the exposure. Each compound was exposed at a minimum of three biological replicates to ensure the reproducibility of the transcriptomic signatures observed. We selected a chemically and structurally diverse set of antibiotics to get the largest spread of transcriptomic responses within a reasonable number of antibiotics. This resulted in the selection of 37 antibiotics comprising the 6 MOA classes, which include DNA replication, RNA synthesis, protein synthesis, cell wall biosynthesis, cell membrane function, and fatty acid synthesis inhibition.

Previous work has highlighted the challenge of evaluating transcriptomic data sets. It is recognized that despite having knowledge of the primary target of each antibiotic, off-target responses, such as the turning on of protective stress response networks, tend to predominate in transcriptomic profiles (19). Due to the convergence of these downstream effects, antimicrobials from separate classes of antibiotics have overlapping gene expression patterns (3). We have observed this phenomenon in our data set, where antibiotics from different MOA classes have overlapping gene profiles; yet, due to the size of our data, set we are able to identify MOA-specific trends from a reduced gene set. Taking this approach, we found 3 major signatures among the 37 transcriptomic profiles when filtering for the top 400 genes with the largest standard deviation on log-fold change values. First, we observed the upregulation of the colanic acid synthesis gene cluster (*wca*), where colanic acid is a special class of capsule polysaccharide that coexpresses with some K or O antigens among E. coli strains (20). The role of colanic acid was unclear for decades, until it was demonstrated that select beta-lactam antibiotics could induce colanic acid formation in E. coli and, in turn, trigger capsule production. We found that although the response is shared across MOAs, genes from the *wca* operon could be favorable candidate markers for identifying antimicrobial activities targeting the Lipid II pathway and peptidoglycan synthesis inhibition by their differential gene expression patterns, although not with absolute fidelity. Additional members of this first gene expression signature included *yjbFG* (b4027 and b4028), *ymgDG* (b1171 and b1172), and *cbrABC* (b3690, b3716, and b3717), which have putative functions in exopolysaccharide biosynthesis, biofilm modulation, and bacteriocin resistance, respectively (21, 22). The RcsAB transcription complex regulates capsule biosynthesis (*wca*), biofilm formation (*yjb*), periplasmic and membrane proteins, and autoregulation of *rcsA*, all of which were observed in our Lipid II cell wall inhibitor transcriptome analysis. Taken together, these results suggest that the observed upregulation in the *wca* gene cluster is a secondary response of the bacterium to antibiotic treatment, as it is regulated by the RcsBC envelope stress response. Despite being a secondary effect of antibiotic treatment, the magnitude (or degree of up or downregulation) in the transcriptomic response of E. coli can help to resolve antibiotics to their associated MOA.

As the majority of the gene expression profiles do not neatly cluster according to MOA, we looked at each MOA in isolation. For the protein synthesis inhibitors, clustering of the overlapping DEGs for PS50 and PS30 inhibitors allowed for the independent sorting of the two subMOAs. The genes in this overlap are mostly upregulated and code for the components of the ribosomal subunits, and the majority of the downregulated genes are associated with various metabolic processes (23). This has been observed before in E. coli, Haemophilus influenzae (24), and Streptococcus pneumoniae (23), where the inhibition of one facet of the ribosomal target will result in an attempt to overcompensate for the loss of the primary target. Conversely, compounds representative of the same MOA do not always have overlapping DEGs when using prescribed fold change and FDR cutoffs. For instance, DS-DG inhibitors have no overlap across the DEGs for the individual transcriptomic profiles. However, the use of all log_2_ fold change (FC) values across all DEGs for this MOA allows for the sorting of the subMOAs. For instance, DEGs of the DS set were enriched for Gene Ontology (GO) terms associated with DNA unwinding, DNA topoisomerase activity, and response to stress; whereas DEGs of the DG set included the genes involved SOS response and response to DNA damage.

Finally, we aimed to find a global gene list that would encompass subMOA resolution within MOA-level resolution. The final model uses 447 genes and predicts the MOA of pure previously observed compounds with 84% accuracy. The incorrect MOA assignments were for the following antibiotics with Pearson correlation coefficient ranging from 0.56 to 0.72 to a profile outside their MOA designations: novobiocin (a DS to RS, rifapentine), polymyxin (a CM assigns to CW, penicillin), monactin (a CM assigns to RS, actinomycin D), trimethoprim (a DS assigns to PS30, doxycycline), and metronidazole (a DS to PS30, doxycycline). The incorrect assignments for novobiocin, polymyxin, and monactin are most likely due to their expression profiles for genes involved with colanic acid biosynthesis. This is apparent due to their high correlation to inhibitors which also upregulate *wcb* genes. The high correlation of trimethoprim and metronidazole to doxycycline, a PS30 inhibitor, is likely due to the shared ability to affect the DNA binding, outer membrane, and metabolism-associated genes within the subset gene list.

For crude extracts from antimicrobial-producing bacterial strains, CW inhibitors are classified incorrectly as CM inhibitors. For crude extracts containing previously unobserved compounds, the classifier model does not perform well (20% accuracy). This highlights the need to expand the training data. We expect that we could improve this resolution by including additional pure compounds and crude extracts of the strains which produce these pure compounds to better simulate noise in the reference data set. However, at the current status, we are able to triage antibiotics and crude extracts to their nearest transcriptome associate MOA bin using the prescribed 447-biomarker gene set. Ultimately, we attained a gene set with remarkable accuracy for determining MOA when considering the existing data set and show that extracts containing known compounds can be rapidly deprioritized. It is, however, clear that MOAs that are “clean,” with single-enzyme targets (DG), are easier to predict than those that have numerous anthropomorphically assigned target groups (DS). Breaking up these artificial categories requires additional experimentation with compounds with defined targets, which is a logical next step.

## MATERIALS AND METHODS

### Strains, media, chemicals, and growth conditions.

The 1× MICs of all antibiotics were determined using the CLSI broth dilution methods. In the MIC assay, 2 μl of an antibiotic dilution series (beginning at 64 μg/ml) was used to treat 100 μl of a culture with an OD_600_ of 0.001 (diluted from cells in exponential growth phase) and then was incubated without shaking for 18 hours before MIC determination. The MIC value is the lowest concentration at which bacterial growth is fully arrested. For antibiotics with greater than 64-μg/ml MICs, we assessed up to 1 mg/ml in 100-μg/ml step-downs.

For the antibiotic challenge experiments, 3 ml of E. coli WO153 (AB1157; *asmB1* Δ*tolC*::*kan*) (https://cgsc2.biology.yale.edu/Strain.php?ID=4509) (8) at an OD_600_ of 0.5, representing mid-log phase, was exposed to each antibiotic in biological triplicate at a 1× MIC for 30 minutes. Due to potential inoculum effects, the MIC for drugs added at an OD_600_ of 0.5 may not be the same as in the MIC assay format; however, all treatments were subject to the same discrepancy. After 30 minutes of exposure to a 1× MIC concentration of an antibiotic, 100 μl of the cells was removed for each exposure to be evaluated for their OD_600_ values and CFU/ml counts. We ensured that the antibiotic-treated sample had an OD_600_ value and CFU/ml counts less than that than of the untreated control. The remainder of the cells were immediately pelleted at 4°C by centrifugation for 10 minutes at 2,000 rpm in 1-ml aliquots. The supernatants were removed, and samples were immediately frozen in liquid nitrogen at −80°C until they were processed for total RNA isolation.

### RNA isolation.

Total RNA was extracted by automation using the NucleoMag RNA extraction kit on the EpMotion robotic liquid handler. For the resulting total RNA, RNA integrity number (RIN) values were obtained to check for RNA quality using the 2200 TapeStation instrument from Agilent Genomics. Acceptable values to proceed to rRNA depletion were above a RIN of 5.

### rRNA depletion.

rRNAs were subtracted from the total RNA to yield only mRNA for library construction using a New England BioLabs (NEB) bacterial rRNA depletion kit at half reactions, with a total RNA input maximum of 400 ng. The rRNA-depleted samples were quality checked using an Agilent bioanalyzer with the Agilent Pico chip for RNA detection for less than 0.5% of rRNA remaining in each sample.

### RNA-seq.

A total of 2 to 5 ng of the rRNA-depleted samples was used as the input material to construct each cDNA library for RNA sequencing using the NEBNext Ultra directional RNA library prep kit from Illumina. The resulting libraries were quality checked using Agilent high-sensitivity DNA chips to ensure proper library size distribution and the absence of small adapters. Libraries were quantified and normalized by quantitative PCR (qPCR) and then sequenced using the NextSeq 500 high-output kit at 150 cycles, producing approximately 9 million, 75-base pair paired-end reads for each library.

### Mapping of RNA-seq data.

Because the laboratory strain WO153 used in this study was derived from the E. coli K-12 strain AB1157 (https://cgsc2.biology.yale.edu/Strain.php?ID=4509) and harbored few genomic differences (asmB1 ΔtolC::kan), a surrogate reference genome from the wild-type E. coli K-12 strain MG1655 (GenBank assembly accession number GCF_000005845.2) was used as the reference genome for read mapping. Adapter trimming was done by AdapterRemoval (v2.1.7) using two identified adapter sequences. Quality trimming was done by Trimmomatic (v0.36). The surviving paired-end (PE) reads were mapped to reference genome of the E. coli K-12 MG1655 strain using BWA-MEM (v0.7.17). Mapped read pairs were counted for each gene using featureCounts (v1.6.1). The focus of this study was the transcriptome response of the E. coli protein-coding genes (see Data Set S8 in the supplemental material). Several regulatory noncoding RNAs have also been shown to be involved in response to antibiotic treatment (25) and will be investigated in future studies.

### Statistical tests.

E. coli exposures were performed at a minimum of three exposures per treatment to obtain statistically significant *P* values and FDRs for each gene. RNA-seq analysis was performed on 219 samples (Data Set S1). All packages used default statistical parameters unless otherwise noted.

### Significance analysis of RNA-seq data.

A total of 219 samples (replicated treatment and control samples) remained after discarding samples with less than 0.4 million read pairs. For the final analysis, 4,007 protein-coding genes were included requiring at least 3 cpm in 3 or more samples. The trimmed mean of M-value (TMM) normalization was used for library size normalization (26). Mean-variance relationship modeling and gene-wise linear modeling were done using limma R package to rank genes in order of evidence for differential expression (27). Differentially expressed genes (DEGs) were selected using |log2(FC)| of >1 and FDR of <0.01 as cutoffs (Data Set S2).

### Network analysis.

Network analysis was performed using Cytoscape v3.7 (9). A total of 3,852 genes that exhibit differential expression in at least 1 treatment were used for network analysis. Edges were defined as their presence or absence in each MOA. The edge-weighted spring-embedded layout in the Cytoscape platform (9) was chosen to create the self-organized network. This algorithm was initially generated by Kamada and Kawai to organize undirected network structures (10). Based on this algorithm, each node is subject to forces that are applied by the edges that act similar to metal springs. We additionally used edge weights which were calculated as a ratio of number of compounds that each DEG was detected over the number of total of compounds that were included in a given MOA (Data Set S3). These edge weights determined the magnitude of forces applied on each node in the network. A total of 14,205 edges were included in the network. For the DEGs that were downregulated, edge weight was multiplied by −1 to indicate negative regulation. For the DEGs that had conflicting regulation within a given MOA (i.e., positive in one compound and negative in other), edge weight was multiplied by 0 to remove the weight and retain the association. The edge-weighted spring-embedded layout was used to visualize the network.

### Unsupervised clustering analysis.

Unsupervised clustering analysis used genes whose fold change values are most different across 37 compounds by selecting 400 genes with the largest standard deviation on log-fold change values. A clustered heatmap of the 37 compounds and 400 genes was generated based on log-fold change values, with a distance metric of the Pearson correlation and the complete linkage clustering method using the “pheatmap” (v1.0.12) R package.

### Visualizations using Venn diagrams and heatmaps.

Each of the 4,007 protein-coding genes for the transcriptomic profiles for each antibiotic were filtered using |log2(FC)| of >1 and FDR of <0.01 as cutoffs. Venn diagrams were visualized using the Bioinformatics and Evolutionary Genomics (BEG) Venn tool (http://bioinformatics.psb.ugent.be/webtools/Venn/). Inputs for the analysis were all DEGs that made these cutoffs for each antibiotic within a MOA class or subMOA. Inputs for DS/DG molecular descriptor analysis were 88 molecular descriptor values obtained using Bioclipse software (25) for the evaluation of 3D .sdf files obtained from PubChem (Data Set S6). Hierarchical clustering used the “hclust” tool (fastcluster v1.1.25) with defaults in R. Heatmaps were generated using the union set of genes for each MOA. The union set includes all genes represented at least in one treatment, with a >2-fold change in transcript level and <1% false discovery rate (FDR) cutoff. A clustered heatmap of the MOA-specific compounds was generated based on log-fold change values with a distance metric of the Pearson correlation and a complete linkage clustering method using the “pheatmap” (v1.0.12) R package.

### GO term enrichment analysis.

Given a list of genes determined by clustering or overlapping differentially expressed gene lists, GO term enrichment analysis was done using the Fisher’s exact test implemented in the “topGO” R package. Terms were collapsed using Revigo (28).

### Correlation/prediction analysis.

In the leave-one-compound-out cross-validation approach, 1 compound is selected as a test compound, and from the remaining 36 compounds, a set of diagnostic genes are selected. The Pearson correlation coefficient is calculated using the log-fold change values between the test compound and each of the remaining 36 compounds. The MOA of the compound closest to the test compound (i.e., the compound with the highest Pearson correlation coefficient) is assigned to the test compound, and the predicted MOA is compared with the known MOA of the test compound. This procedure is repeated for all 37 compounds, and the overall accuracy is estimated by the number of correct predictions.

Three different methods for selecting diagnostic genes were tested, as follows: (i) genes whose fold change values are most different across 37 compounds, (ii) genes that are most significantly up/downregulated in any of 37 compounds, and (iii) genes whose fold change values are most different between MOAs. In method 1, the genes with the largest standard deviation on log-fold change values across 37 compounds were selected. The number of genes from 10 to 4,000 was tested. In method 2, the genes most significantly differentially expressed in terms of FDR were collected from each of the compounds with a fixed number of genes (from 10 to 4,000). In method 3, analysis of variance (ANOVA) was used to test whether log-fold change values are different between any 7 MOAs, and top *n* genes in terms of *P* values were selected for an *n* of 10 to 4,000.

### Crude extract production of antimicrobial-producing microbial strains.

To further test the diagnostic gene sets, strains producing known antibiotics were fermented and the whole broth was processed to produce crude extracts. Strains from the USDA NRRL collection (https://nrrl.ncaur.usda.gov/) were used to produce extracts of valinomycin (Streptomyces roseochromogenes B-1233), bacitracin (Bacillus licheniformis B-1001), penicillin (Streptomyces clavuligerus NRRL 3585), vancomycin (Amycolatopsis orientalis NRRL 2450), novobiocin (Streptomyces spheroids NRRL 2449), erythromycin (Saccharopolyspora erythraea B-24071), and rifampin (Amycolatopsis mediterranei B-3240). Strains from NovoBiotic Pharmaceuticals internal collection were used to produce extracts of teixobactin (Eleftheria terrae L3383), chloramphenicol (*Streptomyces* sp. strain X4251), and actinomycin D (Streptomyces canus C4087).

Strains were inoculated from a frozen glycerol stock onto SMSR4 agar plates (0.125 g casein, 0.1 g potato starch, 1 g Casamino Acids, 100 ml R4 fermentation medium, and 20 g Bacto agar in 1 liter water). Morphology was confirmed under ×10 magnification using a Zeiss Stemi 2000 microscope, and cultures inoculated into 20 ml of Modsb (15 g glucose, 10 g malt extract, 10 g soluble starch, 2.5 g yeast extract, 5 g Casamino Acids, and 0.2 g CaCl_2_-2H_2_O per 1 liter deionized H_2_O [pH 7.0]) in a 250-ml flask, shaken at 150 rpm at 28°C for 2 to 5 days. Upon robust growth, the biomass was diluted 1:20 into 500 ml of production medium R4 (10 g glucose, 1 g yeast extract, 0.1 g Casamino Acids, 3 g proline, 10 g MgCl_2_-6H_2_O, 4 g CaCl_2_-2H_2_O, 0.2 g K_2_SO_4_, 5.6 g *N*-tris(hydroxymethyl)methyl-2-aminoethanesulfonic acid [TES]-free acid [2-[[1,3-dihydroxy-2-(hydroxymethyl)propan-2-yl]amino]ethanesulfonic acid] per 1 liter deionized H_2_O [pH 7]) for all strains except X4251. X4251 was diluted 1:20 into 500 ml of production medium (20 g glucose, 10 g organic soy flour [Bob’s Red Mill], 10 g pharmamedia [Traders Protein], 1 g (NH_4_)_2_SO_4_, 10 g CaCO_3_, and 20 g glycerol per 1 liter deionized H2O). Activity was monitored by bioassay, and the active cultures were harvested between 4 and 7 days of growth in the production medium at 150 rpm at 28°C. Crude extracts were generated by extracting the whole broth culture with an equal volume of water-saturated n-butanol for 3 h at room temperature and sonicating the culture in a water bath for 20 min prior to clarifying the butanol/aqueous layers with centrifugation. The n-butanol layers were removed into clean tubes and dried in a Savant Speedvac concentrator heated to 45°C under vacuum. The dried substances were reconstituted and concentrated in 100% dimethyl sulfoxide (DMSO) at 10× the original volume. Crude extracts were divided into 500-μl aliquots, tested for MIC against WO153, and kept frozen until used for exposures to produce transcriptomes. The production of known compounds was confirmed with mass spectrometry, high-performance liquid chromatography (HPLC) retention time, and/or spectrum of activity against resistant and sensitive test strains. Crude extracts were shipped on dry ice from NovoBiotic Pharmaceuticals to J. Craig Venter Institute overnight.

### Data availability.

The data are available under NCBI BioProject number PRJNA532938.

## Supplementary Material

Supplemental file 1

Supplemental file 2

Supplemental file 3

Supplemental file 4

Supplemental file 5

Supplemental file 6

Supplemental file 7

Supplemental file 8

Supplemental file 9
