# Supplementary material for: Mechanism-of-Action Classification of Antibiotics by Global Transcriptome Profiling
Source: Antimicrob Agents Chemother. 2020 Feb 21;64(3):e01207-19. doi: 10.1128/AAC.01207-19 (PMC7038283; doi:10.1128/AAC.01207-19)
Supplement: Supplemental file 1 [file AAC.01207-19-s0001.pdf]

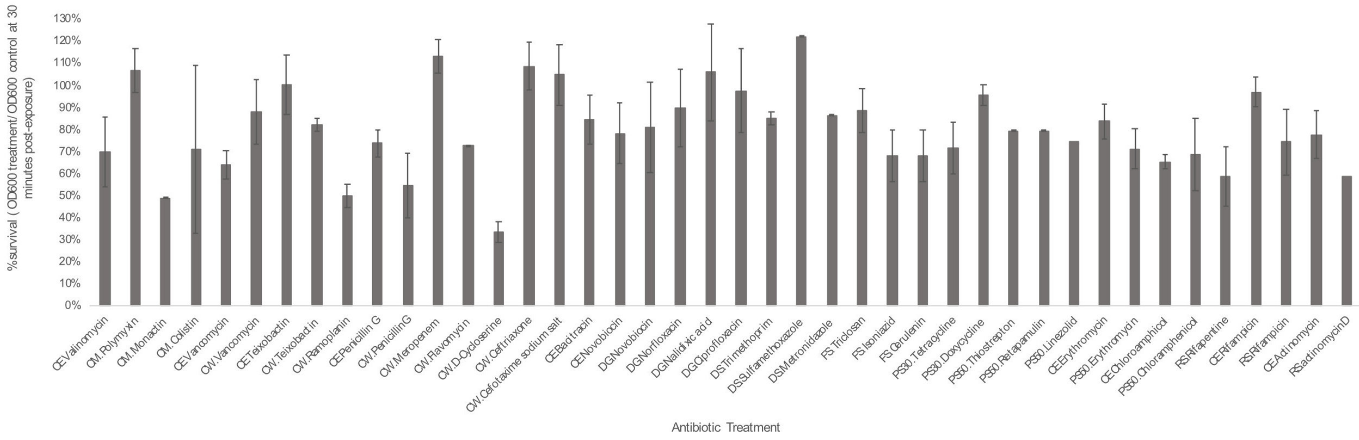

FIG S1. % survival of antibiotic treated samples compared to 30 minute solvent controls. Error bars are standard deviations taken from 3 biological replicates with 3 technical replicates each.

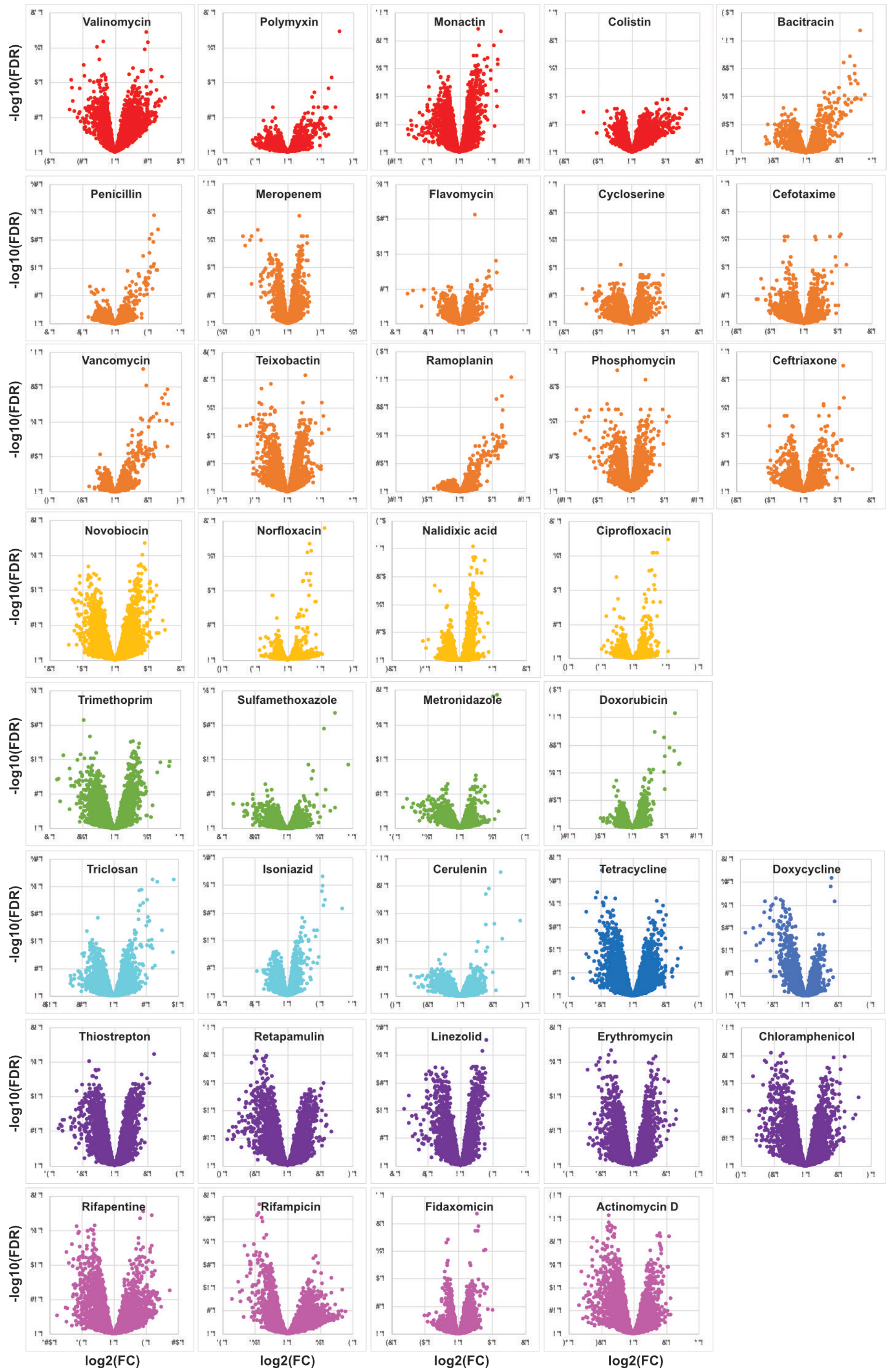

FIG S2. Volcano plots for each treatment are presented. X-axis values are $\log_2$(FC) and Y-axis values are $-\log_{10}$(FDR). Volcano plots were colored according to the MOA as follows: CM (red), CW (orange), DG (yellow), DS (green), FAS (cyan), PS30 (blue), PS50 (purple) and RS (pink).

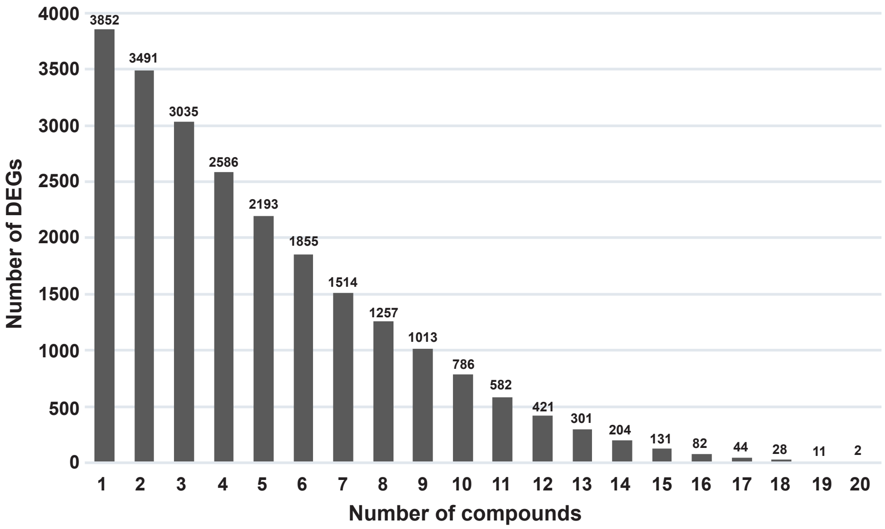

FIG S3. Number of DEGs were plotted as a bar graph based on the number of treatments (compounds) they were identified with $|\log2(FC)|>1$ and $FDR<0.01$ as a cutoff.

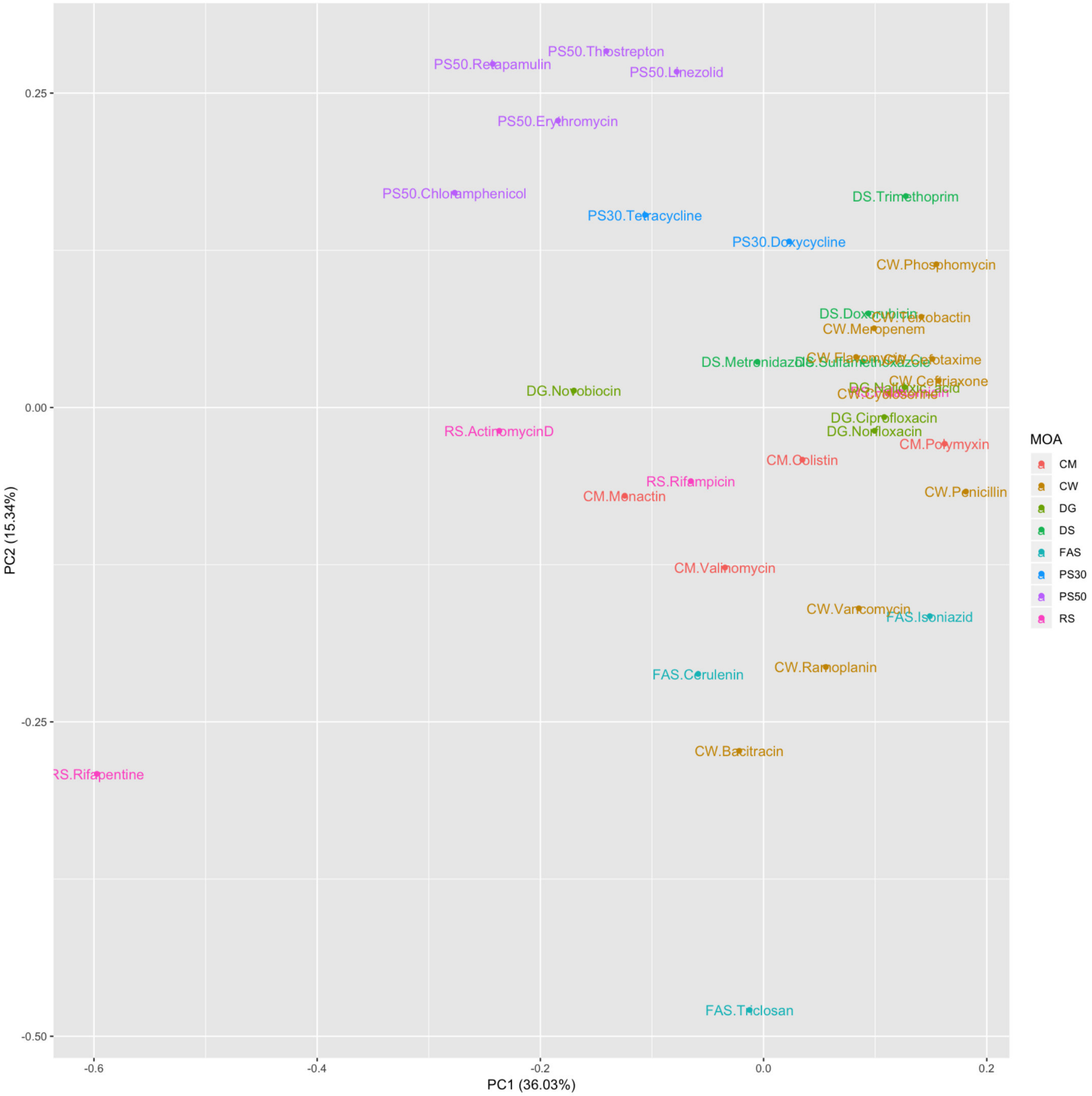

FIG S4. Principle component analysis (PCA) plot based on $\log_2$FC values for all 4007 genes for all treatments.

										Lipid II				Lipid II and III	Cell wall precursors		beta-lactams							
Gene	locus_tag	RS.Rifapentine	RS.Rifampicin	RS.Fidaxomicin	RS.ActinomycinD	DG.Novobiocin	DG.Norfloxacin	DG.Nalidixic_acid	DG.Ciprofloxacin	CW.Vancomycin	CW.Bacitracin	CW.Ramoplanin	CW.Flavomycin	CW.Teixobactin	CW.Phosphomycin	CW.Cycloserine	CW.Penicillin	CW.Meropenem	CW.Ceftriaxone	CW.Cefotaxime	CM.Valinomycin	CM.Polymyxin	CM.Monactin	CM.Colistin
wza	b2062	8.0	1.2	-0.2	3.0	2.0	0.7	0.1	0.1	4.9	6.1	6.6	4.4	1.4	0.2	0.8	2.9	1.4	-0.5	0.1	0.9	1.1	2.4	-0.6
wzb	b2061	8.1	1.3	0.0	1.8	1.7	1.1	-0.4	0.7	4.6	6.1	6.7	3.6	1.2	-1.7	0.5	2.7	1.6	-0.8	-0.2	0.5	0.8	1.6	-2.1
wzc	b2060	7.7	1.2	-0.1	1.9	1.7	0.6	0.2	0.7	4.8	5.9	6.6	3.5	1.3	-0.7	0.6	3.2	1.1	-0.3	0.0	1.0	1.5	1.9	-0.1
wcaA	b2059	8.1	1.0	0.1	1.5	2.0	0.6	0.5	0.3	4.3	5.5	6.2	2.9	1.1	-0.4	0.7	3.1	1.4	-0.5	0.1	0.8	2.1	1.8	0.0
wcaB	b2058	8.4	1.5	0.1	1.5	2.1	0.8	0.0	0.3	4.7	5.4	6.3	2.7	1.5	0.7	0.9	3.8	1.6	0.6	-0.3	1.2	2.6	1.8	0.5
wcaC	b2057	7.0	0.9	0.0	1.8	2.0	0.6	-0.2	0.1	3.9	4.9	5.9	3.2	0.8	-1.0	0.8	2.8	1.2	-0.1	0.4	0.7	1.8	1.5	0.8
wcaD	b2056	7.2	1.1	-0.1	2.1	1.4	0.5	-0.2	0.1	3.8	4.2	5.6	2.7	0.7	-1.4	1.0	2.7	1.3	-1.1	-0.4	0.9	1.4	1.2	0.5
wcaE	b2055	6.9	0.7	-0.4	1.7	1.7	1.0	0.0	0.4	4.2	4.3	5.8	3.1	0.5	-0.9	0.4	2.8	1.2	-0.5	0.1	0.4	1.1	1.4	-0.2
wcaF	b2054	7.8	1.4	0.1	1.6	2.0	0.7	0.2	0.7	3.9	4.3	5.8	3.1	0.7	-0.1	0.1	2.8	1.4	-0.4	0.1	0.3	1.7	1.7	-0.1
gmd	b2053	7.4	0.7	0.1	1.3	2.1	0.4	-0.2	0.2	4.3	4.9	5.9	3.4	1.4	-0.3	1.3	3.6	1.4	-0.1	0.1	0.2	2.3	1.2	-0.4
wcaG	b2052	7.2	0.9	0.1	1.1	2.3	0.4	-0.2	-0.1	4.0	4.7	5.9	3.5	1.5	-0.5	1.1	3.5	1.5	0.0	0.5	0.5	2.4	1.5	-0.4
wcaH	b2051	7.8	1.0	0.1	0.7	1.6	0.5	-0.1	0.0	3.5	4.3	5.6	3.0	1.5	-0.3	1.2	3.0	1.4	-0.5	-0.6	0.3	1.7	1.4	0.0
wcaI	b2050	7.5	0.9	0.0	1.2	2.1	0.3	-0.2	-0.2	3.5	4.5	5.5	3.0	1.0	-0.2	1.2	3.1	1.4	0.0	0.2	0.3	2.0	1.3	-0.1
cpsB	b2049	6.9	0.9	0.1	0.9	2.1	0.2	-0.2	-0.2	3.5	4.0	5.6	2.9	0.7	0.3	1.3	3.3	1.1	0.0	0.3	0.8	2.3	0.9	0.1
cpsG	b2048	7.0	0.8	-0.1	0.6	1.9	0.2	-0.3	0.1	3.4	3.9	5.4	2.7	0.7	-0.4	1.3	3.1	0.8	-0.1	0.4	0.2	2.1	1.0	-0.1
wcaJ	b2047	6.9	1.3	0.0	0.7	2.0	0.1	0.1	0.1	2.8	3.7	5.1	2.5	0.5	-0.3	1.4	2.8	0.8	-0.2	0.1	1.3	2.0	0.9	0.3
wzxC	b2046	7.2	1.3	-0.1	0.9	2.0	0.2	0.0	0.1	2.0	3.1	4.7	2.0	0.6	-0.7	1.0	2.2	0.8	-0.2	0.0	1.2	1.6	0.9	0.8
wcaK	b2045	6.6	0.5	0.0	-0.9	1.6	0.0	-0.3	0.0	1.7	3.0	4.3	1.4	0.5	-0.4	1.2	2.3	0.6	-0.4	-0.3	0.3	1.3	0.0	-0.1
wcaL	b2044	4.6	0.6	-0.1	0.9	1.3	0.0	-0.2	-0.3	0.9	1.8	3.2	0.7	0.3	0.3	0.7	1.2	0.2	-0.4	0.0	0.6	1.1	-0.2	0.6
wcaM	b2043	5.5	1.1	0.1	0.3	1.2	-0.5	-0.4	-0.3	0.9	1.7	2.9	0.5	0.2	0.2	1.0	1.3	-0.1	-0.1	0.0	0.8	1.4	-0.1	0.0
wcaN	b2042	1.3	0.0	0.0	1.4	-0.5	-0.4	-0.1	-0.3	0.5	0.9	1.4	0.0	-0.1	0.3	0.4	0.3	-0.3	-0.2	0.1	-0.4	-0.1	-0.6	-0.2

FIG S5. Expression of the colanic acid capsule exopolysaccharide gene cluster (*wca*) for Lipid II inhibitory antimicrobial compounds (ramoplanin, vancomycin, and bacitracin) and in flavomycin (CW), polymyxin (CM), monactin (CM), rifapentine (RS), novobiocin (DG). No activation was detected in antimicrobials targeting inhibition of protein synthesis, DNA synthesis, or fatty acid synthesis. Log2 fold-change shown by red bars (0-10). FDR shown by colored circles: <0.05 (yellow), <0.01 (red).

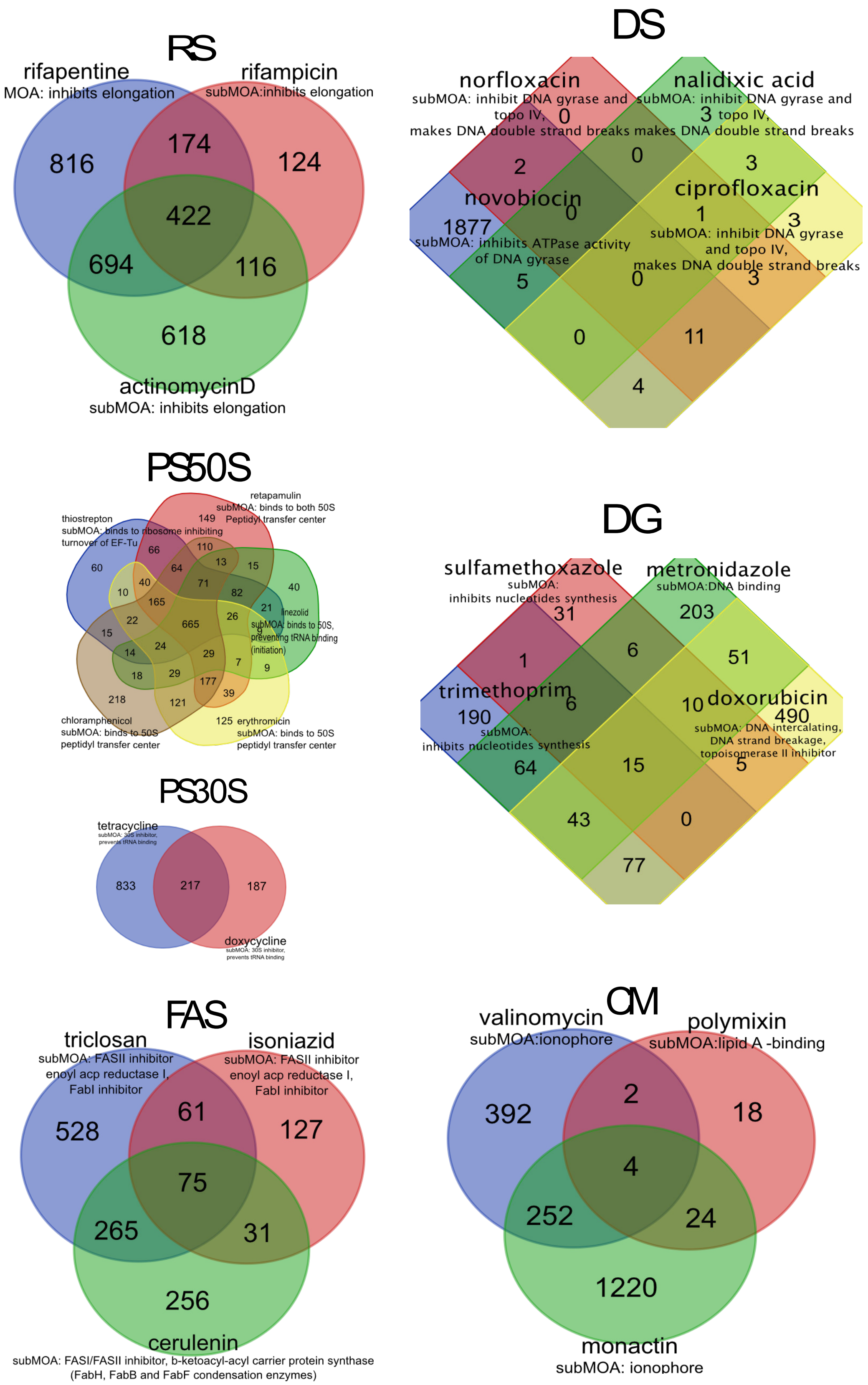

FIG S6. Venn Diagrams for union sets of DEGs to show subMOA DEG overlap.

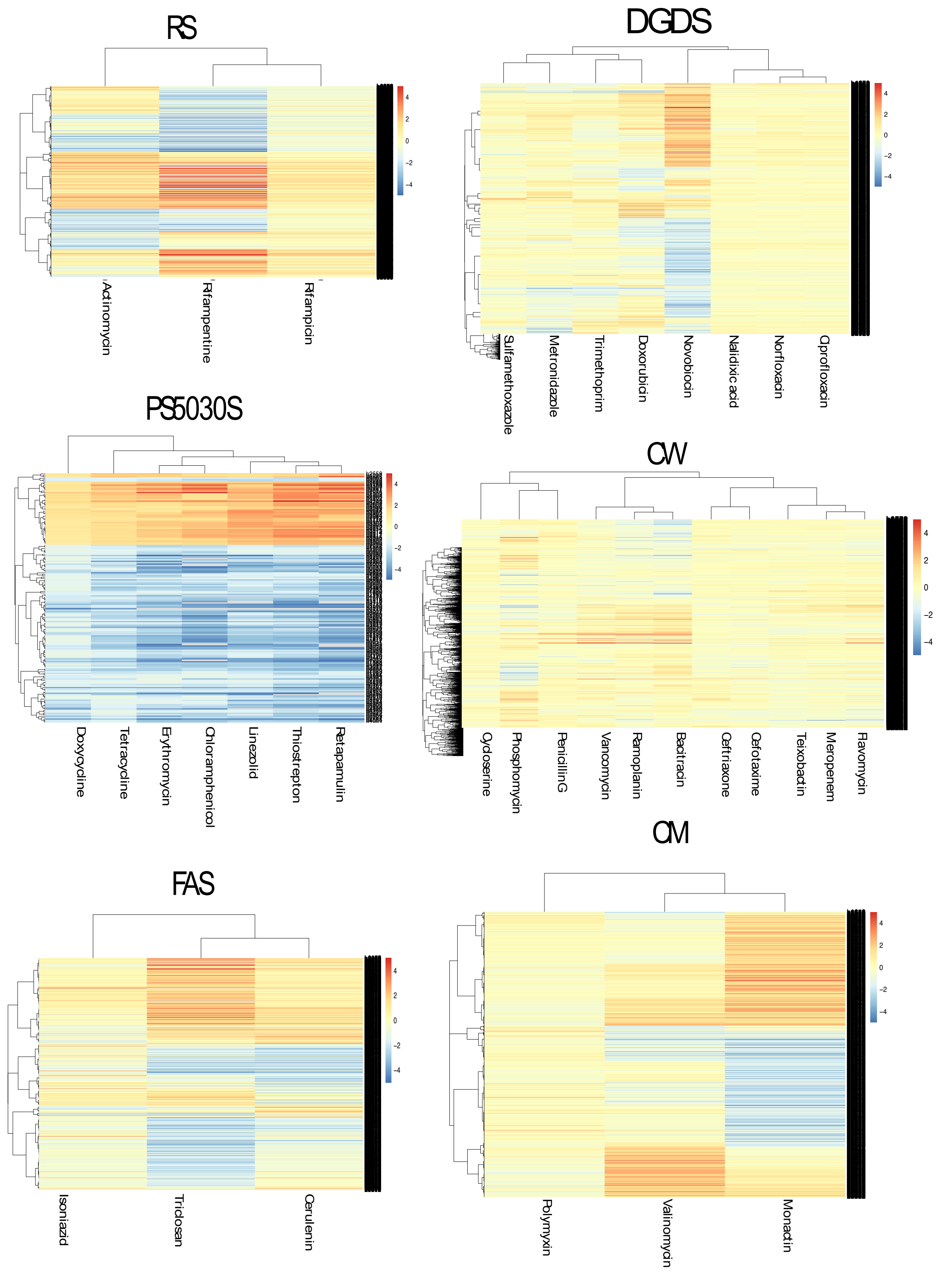

FIG S7. Heatmaps for union sets of DEGs to show subMOA DEG hierarchical clustering.

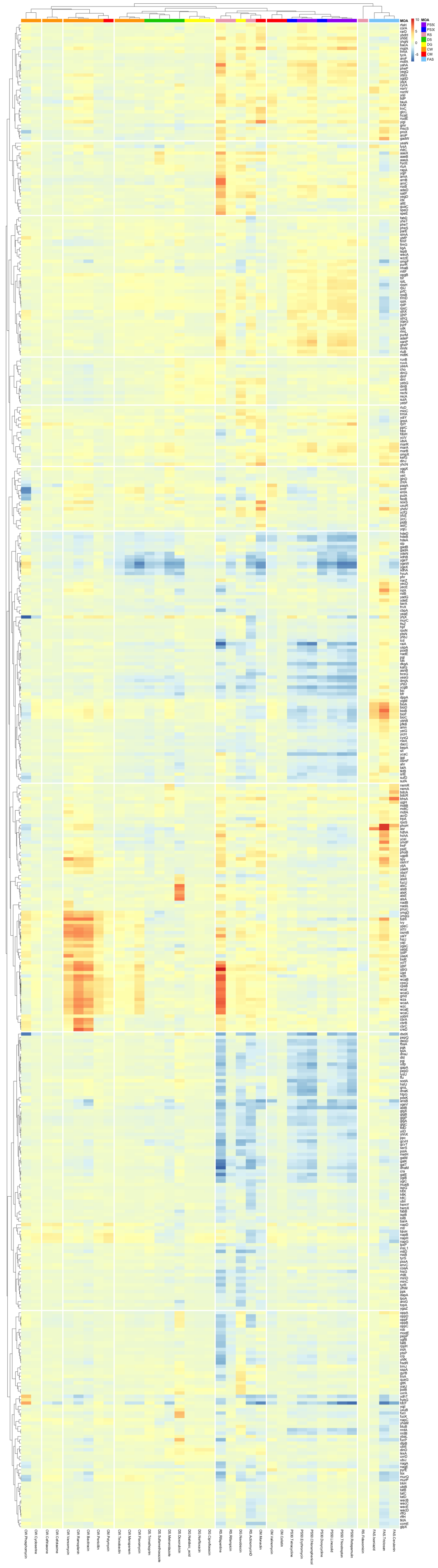

FIG S8. Hierarchical clustering of the 447-biomarker gene set resolves the antibiotics into their classical MOA designations.

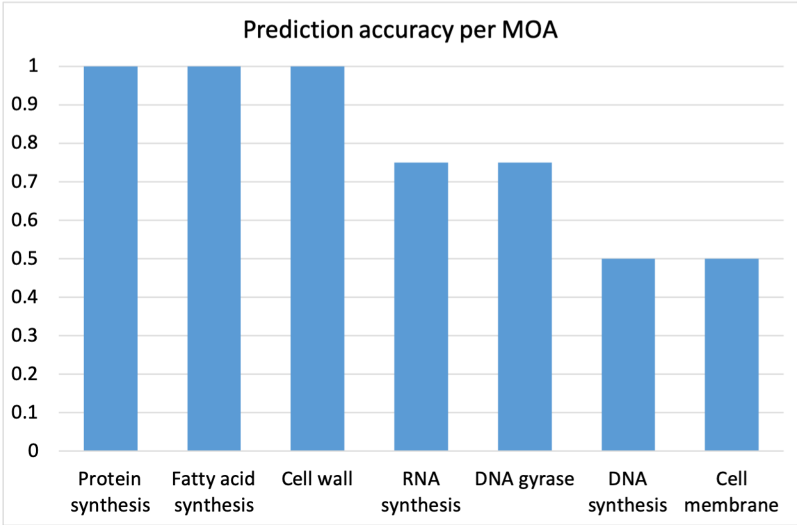

FIG S9. Correlation-based prediction accuracy per MOA for antimicrobial reference compounds by leave-one-compound-out cross-validation using a 447-biomarker gene set.
